# First-Year Mortality in Infants Born Moderately Preterm and Late Preterm: A National Register Study

**DOI:** 10.1016/j.jpedcp.2026.200206

**Published:** 2026-03-24

**Authors:** Kati Kallio, Riitta Ojala, Tiina Luukkaala, Outi Tammela

**Affiliations:** 1 Doctoral Program in Medicine, Tampere University, Tampere, Finland; 2 Department of Pediatrics, Oulu University Hospital, Oulu, Finland; 3 Tampere Center for Child Health Research, Tampere University, Tampere, Finland; 4 Department of Pediatrics, Tampere University Hospital, Tampere, Finland; 5 Research, Development, and Innovation Center, Tampere University, Tampere, Finland; 6 Faculty of Health Sciences, Tampere University, Tampere, Finland

**Keywords:** moderately preterm, late preterm, early neonatal mortality, late neonatal mortality, postneonatal mortality, causes of death, predictors of mortality

## Abstract

**Objective:**

To investigate first-year mortality and predictors and causes of death in infants born moderately preterm (MP; birth at 32^0/7^-33^6/7^ weeks of gestation) and late preterm (LP; 34^0/7^-36^6/7^) compared with infants born very preterm (<32^0/7^) and term (≥37^0/7^-41^6^^/7^) and to establish mortality rates by region, hospital level, and time.

**Study design:**

Data on all infants (n = 1 546 787) born in Finland between 1991 and 2016 were collected from national registers. Of those born prematurely (5.9%), infants born MP and LP accounted for 85,9%.

**Results:**

Early, late, and postneonatal mortality decreased with advancing gestational age. Despite overall declines, early neonatal mortality remained significantly greater in infants born MP and LP than in infants born at term. Over time, early neonatal mortality decreased only in MP and LP groups, whereas first-year mortality declined in infants born MP and LP. Predictors of death in all mortality categories in infants born MP and LP included small for gestational age, low Apgar score, and ventilator treatment. Some regional and hospital-level differences in early neonatal mortality were observed. Causes of death in infants born MP and LP resembled those of term more than infants born very preterm, with congenital anomalies being the most common, albeit not easily preventable causes of death.

**Conclusions:**

Infants born MP and LP had an elevated risk of early neonatal mortality compared with infants born at term. Continued reductions may be achieved by targeting preventable causes such as asphyxia and respiratory distress, while counseling-based interventions may help prevent sudden infant death syndrome and reduce late neonatal and postneonatal mortality.

Preterm birth (birth at <37 weeks of gestation) is a leading cause of mortality among children younger than 5 years old.^1^ It has been estimated globally that 13.4 million infants were born preterm in 2020 (9.9% of live births).^2^ Infants born moderately preterm (MP; 32^0/7^-33^6/7^ weeks of gestation) and late preterm (LP, 34^0/7^-36^6/7^ weeks of gestation)^3^ account for more than 80% of infants born prematurely.^4^ Only 2 previous studies, with US and Canadian birth cohorts,^5^^,^^6^ have investigated infants born preterm in the early neonatal, late neonatal, and postneonatal mortality categories, and they concluded that being born even slightly preterm increases the risk of death. Both studies included only live-born singletons more than 20 years ago. They also did not look for predictors of mortality in any of the categories. To update this information, more recent studies on birth populations are needed.

We found earlier that infants born MP and LP have better neurodevelopmental^7^ and respiratory^8^ outcomes than infants born very preterm (VP) but worse outcomes compared with infants born at term. Here our aim was to investigate early neonatal, late neonatal, and postneonatal mortality, predictors of mortality, and causes of death in infants born MP and LP in comparison with infants born VP (<32^0/7^ weeks of gestation) and at term (≥37^0/7^ -41^6^^/7^ weeks of gestation).

We hypothesized that early neonatal, late neonatal, and postneonatal mortality rates would decrease with gestational age and would be lower in infants born MP and LP than in infants born VP but greater than in infants born at term. We also aimed to establish whether these mortality rates differ by region, hospital level, and/or by time.

## Methods

The study population included infants born in Finland between 1991 and 2016 (n = 1 546 787). Data were obtained from the Finnish Medical Birth Register (MBR), which is maintained by the National Institutes of Health and Welfare. The MBR comprises information on live births and stillbirths with a gestational age of 22^0/7^ weeks or more and/or a birth weight of 500 g or more. Gestational age was determined by an ultrasound scan taken in early pregnancy and was adjusted when the ultrasound estimate differed by 5-7 days or more from the gestational age on the basis of the last menstrual period. MBR includes not only data on mothers' interventions, care, and diagnoses during pregnancy and delivery but also information on infants' neonatal care and diagnoses during the first week of life or until hospital discharge. The National Very Preterm Infant Register was established as part of the MBR in 2004 and contains medical data on infants with birth weights of less than 1501 g and those born at less than 32^0/7^ weeks of gestational age. Therefore, data from this register also were included in the study. In addition, the National Institutes of Health and Welfare maintains 2 registers in which the diagnoses, surgical treatment, and hospital care of mothers and newborns are recorded. Before 1994, these data were collected in the Hospital Discharge Register; thereafter, they were included in the Care Register for Health Care. From 1998 onwards, the Care Register for Health Care also included data on outpatient visits.

Mortality during the first year of life was categorized into early neonatal mortality, defined as deaths of live-born infants within 0-6 days, late neonatal mortality (death between 7 and 27 days), and postneonatal mortality (death between 28 and 365 days). We excluded stillbirths and infants born post-term (≥42^0/7^ weeks of gestation). Data on deaths were obtained from the Cause of Death Register from Statistics Finland. Between 1991 and 1995, diagnoses were recorded according to the *International Classification of Diseases*, *Ninth Revision* (ICD-9), after which they were based on the *International Classification of Diseases*, *Tenth Revision* (ICD-910).^9^ Thus, the ICD-9 codes were reclassified to match the ICD-10 diagnoses. For causes of death, we used the codes entered by clinicians as the leading causes of death. We selected the leading cause of death because it represents the primary reason for death. We anticipated that numerous contributing causes might be recorded, and therefore, to maintain clarity and consistency in the analysis, we focused exclusively on the underlying cause.

The data were analyzed over the following 4 time periods: 1991-1995, 1996-2001, 2002-2008, and 2009-2016. These periods were selected because the disease classification system changed from ICD-9 to ICD-10 in 1996, and the MBR revised its data collection forms on October 1, 1990, and January 1, 1996. The infants were categorized into 4 subgroups according to their gestational age: VP, MP, LP, and term. The delivery units were classified according to the level of care: university hospital (level III), central hospital (level II), or other places of delivery (regional hospital, health center, home birth). Regional differences were evaluated among 5 university hospital districts (A, B, C, D, and E) in Finland.

As predictors of mortality, we sought maternal and newborn-related factors. Maternal factors included mother's age, mother's diabetes (information available from 1991 to 2004, after which diabetes mellitus was determined using ICD-10 code O24), smoking during pregnancy, antenatal corticosteroids (both available from 2004), parity, number of fetuses, and mode of delivery. As newborn-related risk factors, we included birth weight; sex; 1-minute Apgar score; need for resuscitation; admission to a neonatal unit, including intensive care units and special baby care units; antibiotic treatment; and ventilator treatment. Ventilator treatment included invasive mechanical ventilation, and resuscitation was defined as chest compressions and/or intubation performed in the delivery room. Antibiotic treatment was recorded if initiated during the first week of life. Infants who were small for gestational age (SGA) were defined as those with a birth weight of less than 2 SDs below the mean birth weight for gestational age, whereas infants who were large for gestational age were defined as those with a birth weight of greater than 2 SDs over the mean weight for gestational age, according to Finnish gender-specific fetal growth curves.^10^

### Statistical Analysis

Infants born stillborn (n = 5533) and post-term (n = 69 805) were excluded from the analyses. Gestational age was unknown among 6173 children. The data size was after exclusions 1 465 350 live births. A multivariable-adjusted logistic regression model was used to analyze early neonatal, late neonatal, and postneonatal mortality. The results are shown by number of deceased infants (n) with percentages and by ORs with 95% CIs. The mortality rates of the MP and LP gestational age groups compared with the term group are expressed using multivariable-adjusted hazard regression analyses that show results by hazard ratios (HRs) with 95% CIs. The change in first-year mortality during the follow-up period has been modeled using multivariable-adjusted Poisson regression with survival time as an offset variable. The results were expressed by incidence rate ratios with 95% CI. Statistical analyses were conducted using IBM SPSS Statistics, version 29.0.1.0. Two-tailed *P* values <.001 were considered statistically significant.

## Results

During the study period, 5.9% of the children were born prematurely. Of all the children, 11 506 (0.75%) were born moderately preterm and 62 890 children (4.3%) were born late preterm. The MP and LP groups comprised 85.9% of the preterm group. The characteristics of the infants and their mothers are shown in Table I.

**Table I:** Characteristics of live-born children (n = 1 465 350)

Characteristics	VP (<32^0/7^ wk) n = 12 155		MP (32^0/7^-33^6/7^ wk) n = 11 056		LP (34^0/7^-36^6/7^ wk) n = 62 890		FT (37^0/7^-41^6/7^ wk) n = 1 379 249	
Mother								
Age, y, mean (SD)	30.3	(5.8)	30.0	(5.7)	29.8	(5.6)	29.4	(5.3)
Diabetes,∗ No. (%)	526	(4.3)	767	(6.9)	5353	(8.5)	68 910	(5.0)
Smoking, after first trimester, No. (%)								
Nonsmoker	9164	(75)	8746	(79)	50 594	(80)	1 142 218	(83)
Quit	370	(3.0)	359	(3.2)	2146	(3.4)	47 280	(3.4)
Smoked	1820	(15)	1570	(14)	8378	(13)	157 980	(12)
Missing	801	(6.6)	381	(3.4)	1772	(2.8)	31 771	(2.3)
Earlier deliveries, No. (%)								
Primipara††								
No	5917	(49)	5082	(46)	32 020	(51)	829 122	(60)
Yes	6198	(51)	5969	(54)	30 826	(49)	549 357	(40)
No. of fetuses								
1	8665	(71)	7579	(69)	49 114	(78)	1 355 141	(98)
2	3160	(26)	3110	(28)	13 381	(21)	24 087	(1.7)
≥3	330	(2.7)	367	(3.3)	395	(<1)	21	(<1)
Assisted reproductive technology†	1086	(10)	1150	(10)	4268	(6.8)	29 472	(2.1)
Place of birth								
University hospital (level III)	9967	(82)	6833	(62)	27 851	(44)	442 836	(32)
Central hospital (level II)	2072	(17)	4120	(37)	27 756	(44)	646 019	(47)
Other	115	(0.9)	103	(0.9)	7283	(12)	290 392	(21)
Mode of delivery††								
Vaginal	4870	(40)	5090	(46)	41 389	(66)	1 171 869	(85)
Elective cesarean delivery	1342	(11)	1671	(15)	6921	(11)	100 960	(7.3)
Nonelective caesarean delivery	5916	(49)	4285	(39)	14 544	(23)	105 662	(7.7)
Antenatal steroids‡	3840	(32)	2841	(26)	5302	(8.4)	7219	(0.5)
University hospital district††								
A	4239	(35)	3625	(33)	21 137	(34)	477 051	(35)
B	1724	(14)	1597	(14)	9097	(15)	175 731	(13)
C	2461	(20)	2318	(21)	13 827	(22)	306 154	(22)
D	1878	(16)	1815	(16)	9205	(15)	203 047	(15)
E	1848	(15)	1701	(15)	9622	(15)	217 058	(16)
Newborn, No. (%)								
Boys	6648	(55)	6106	(55)	34 296	(55)	701 432	(51)
Gestational day§								
AGA	11 414	(94)	10 351	(94)	58 375	(93)	1 307 813	(95)
SGA	473	(3.9)	438	(4.0)	2292	(3.6)	34 387	(2.5)
LGA	268	(2.2)	267	(2.4)	2223	(3.5)	37 049	(2.7)
Apgar 1 min								
7-10	5288	(44)	8344	(76)	56 128	(89)	1 321 753	(96)
0-6	6655	(55)	2545	(23)	6437	(10)	55 426	(4.0)
Unknown	212	(1.7)	167	(1.5)	325	(0.5)	2070	(0.2)
Resuscitation¶	4031	(33)	1119	(10)	1620	(2.6)	5751	(0.4)
Ventilator treatment∗∗	6813	(56)	2202	(20)	2941	(4.7)	5786	(0.4)
Antibiotic treatment	8666	(71)	5406	(49)	9237	(15)	42 651	(3.1)
Study period								
1991-1995	2526	(21)	2218	(20)	12 694	(20)	289 755	(21)
1996-2001	2886	(24)	2602	(24)	14 565	(23)	309 328	(22)
2002-2008	3235	(27)	2954	(27)	16 319	(26)	360 519	(26)
2009-2016	3508	(29)	3282	(30)	19 312	(31)	419 647	(30)

*AGA*, appropriate for gestational age; *HC*, head circumference; *LGA*, large for gestational age; *SDd*, standard deviation derived from the data.

∗ Data on diabetes were available from 1991 to 2004. After 2004, diabetes mellitus was determined using ICD-10 code O24.

† Assisted reproductive technology: insemination, in vitro fertilization, and others.

‡ Data on antenatal steroids were available from 2004 to 2016.

§ Gestation age was defined according to https://pubmed.ncbi.nlm.nih.gov/23768051/ when the calculation of variation was based on SDs, which were calculated from the weight, length, and HC data in all subgroups (SDd).

¶ Ventilator treatment included invasive mechanical ventilation.

∗∗ Resuscitation was defined as chest compressions and/or intubation performed in the delivery room.

†† Indicates the number of the unknowns <1% in all term groups, not presented separately.

### Mortality Rates

Early neonatal, late neonatal, and postneonatal mortality rates in the whole population are presented in Supplementary Table 1 (available at www.jpeds.com) and only among the MP and LP groups in Table II. The greatest proportion of deaths occurred during the first 6 days of life in all preterm groups, but in the infants born at term, most deaths occurred after the early neonatal period. The MP and LP groups accounted for 19.3% of early neonatal, 16.9% of late neonatal, and 16.9% of postneonatal mortality. Of the live-born infants, 2.5% (n = 276 of 11 056) in the MP group and 0.98% (n = 617 of 62 890) in the LP group, compared with 15.8% (n = 1912) in the VP group and 0.15% (n = 2088) in the term group died before 1 year of age. Multivariable-adjusted (adjusting factors were shown in Table II) first-year mortality was statistically significantly (*P* < .001) greater in the MP group (HR 4.61, 95% CI 3.98-5.34) and in LP group (HR 3.48, 95% CI 3.14-3.86) compared with the term group.

**Table II:** Early neonatal, late neonatal, and postneonatal mortality rates among infants born MP and LP

Characteristics	No.	Early neonatal mortality (0-6 days) n = 536 (0.7%) of n = 73 946			Late neonatal mortality (7-28 days) n = 116 (0.2%) of n = 73 410			Post neonatal mortality (28-365 days) n = 241 (0.3%) of n = 73 294		
		n	OR	(95% CI)	n	OR	(95% CI)	n	OR	(95% CI)
Gestation weeks										
MP (32^0/7^-33^6/7^)	11 506	188	**1.74**	**(1.39-2.17)**	28	0.65	(0.41-1.03)	60	0.92	(0.66-1.27)
LP (34^0/7^-36^6/7^)	62 890	348	1.00		88	1.00		181	1.00	
Mother										
Age, y										
20-40	69 765	499	1.00		108	1.00		211	1.00	
<20	2002	13	0.95	(0.52-1.73)	5	1.51	(0.59-3.87)	15	2.03	(1.16-3.54)
>40	2179	24	0.96	(0.60-1.55)	3	0.59	(0.18-1.95)	15	1.81	(1.05-3.10)
Diabetes∗										
No	67 856	519	1.00		108	1.00		225	1.00	
Yes	6090	20	0.53	(0.33-0.85)	8	0.63	(0.28-1.41)	16	0.79	(0.46-1.36)
Smoking after first trimester										
Nonsmoker	59 340	425	1.00		88	1.00		166	1.00	
Quit	2505	14	1.06	(0.60-1.87)	2	0.63	(0.15-2.59)	9	1.46	(0.73-2.89)
Smoked	9948	62	0.70	(0.52-0.93)	20	1.13	(0.68-1.89)	59	**1.82**	**(1.34-2.48)**
Missing	2153	35	1.51	(0.99-2.31)	6	1.63	(0.69-3.84)	7	1.05	(0.49-2.25)
Primipara∗										
No	37 102	330	1.00		66	1.00		141	1.00	
Yes	36 795	198	**0.56**	**(0.46-0.68)**	50	0.68	(0.46-0.99)	100	**0.63**	**(0.48-0.83)**
Number of fetuses										
1	56 693	474	1.00		95	1.00		202	1.00	
2	16 491	59	**0.38**	**(0.28-0.51)**	21	0.80	(0.49-1.31)	37	0.65	(0.45-0.93)
≥3	762	3	0.60	(0.18-2.00)	0			2	0.76	(0.18-3.17)
Antenatal steroids										
No	65 803	482	1.00		101	1.00		210	1.00	
Yes	8143	54	0.86	(0.60-1.24)	15	0.86	(0.44-1.66)	31	0.95	(0.60-1.49)
Place of birth										
University hospital	34 684	348	1.00		66	1.00		137	1.00	
Central hospital	31 876	163	**0.53**	**(0.43-0.66)**	45	0.76	(0.50-1.15)	87	0.78	(0.58-1.04)
Other	7386	25	**0.34**	**(0.22-0.53)**	5	0.78	(0.30-2.04)	17	0.94	(0.55-1.62)
Mode of delivery∗										
Vaginal	46 479	262	1.00		45	1.00		124	1.00	
Elective cesarean delivery	8592	65	0.65	(0.47-0.90)	22	1.34	(0.77-2.33)	33	0.98	(0.65-1.48)
Nonelective caesarean delivery	18 829	200	**0.61**	**(0.49-0.76)**	49	0.98	(0.63-1.53)	84	0.88	(0.65-1.19)
University Hospital District										
A	24 762	163	1.00		33	1.00		80	1.00	
B	10 694	64	1.02	(0.74-1.41)	12	0.82	(0.41-1.61)	40	1.03	(0.70-1.53)
C	16 145	133	**1.68**	**(1.29-2.19)**	33	1.78	(1.07-2.97)	40	0.79	(0.53-1.17)
D	11 020	86	1.46	(1.08-1.99)	22	1.28	(0.72-2.03)	41	1.07	(0.72-1.60)
E	11 323	90	1.08	(0.80-1.44)	16	0.76	(0.41-1.61)	40	0.79	(0.53-1.17)
Newborn										
Sex										
Boy	40 402	305	1.00		60	1.00		144	1.00	
Girl	33 544	231	0.82	(0.68-0.99)	56	1.15	(0.79-1.68)	97	0.85	(0.66-1.11)
Gestational age										
AGA	68 726	405	1.00		83	1.00		196	1.00	
SGA	2730	107	**5.02**	**(3.85-6.54)**	24	**4.63**	**(2.82-7.60)**	39	**3.72**	**(2.56-5.40)**
LGA	2490	24	1.35	(0.84-2.17)	9	2.68	(1.24-5.77)	6	0.76	(0.32-1.77)
Apgar 1 min										
7-10	64 472	95	1.00		44	1.00		141	1.00	
0-6	8982	425	**28.6**	**(22.2-36.7)**	70	**5.07**	**(3.29-7.84)**	95	**3.18**	**(2.36-4.28)**
Missing	492	16	**23.0**	**(12.3-43.1)**	2	3.91	(0.91-16.8)	5	3.70	(1.48-9.24)
Admission to a neonatal unit										
No	33 419	224	1.00		16	1.00		51	1.00	
Yes	40 527	312	**0.32**	**(0.25-0.40)**	100	1.83	(1.00-3.34)	190	1.77	(1.23-2.54)
Resuscitation at birth										
No	71 207	318	1.00		68	1.00		208	1.00	
Yes	2739	218	**6.03**	**(4.69-5.47)**	48	**3.81**	**(2.37-6.13)**	33	1.02	(0.67-1.56)
Ventilator										
No	68 803	331	1.00		55	1.00		162	1.00	
Yes	5143	205	**4.11**	**(3.09-5.47)**	61	**4.63**	**(2.71-7.91)**	79	**3.29**	**(2.27-4.78)**
Antibiotic treatment										
No	59 303	412	1.00		58	1.00		136	1.00	
Yes	14 643	124	**0.22**	**(0.16-0.29)**	58	0.87	(0.54-1.40)	105	1.21	(0.86-1.69)
Study period										
1991-1995	14 912	169	1.00		33	1.00		61	1.00	
1996-2001	17 167	138	0.90	(0.70-1.16)	28	0.82	(0.48-1.37)	57	0.84	(0.58-1.21)
2002-2008	19 273	127	0.83	(0.64-1.08)	23	0.59	(0.34-1.03)	56	0.70	(0.48-1.02)
2009-2016	22 594	102	**0.56**	**(0.41-0.76)**	32	0.79	(0.45-1.40)	67	0.78	(0.52-1.16)

Result (for n < 50) missing cases were not shown.

Logistic regression analyses were used with multivariable adjusted models (n = 75 213). The results are shown by number of deceased infants (n) and ORs with 95% CIs.

Highly statistically significant (*P* < .001) ORs are shown in bold.

Assisted reproductive technology: insemination, in vitro fertilization, and others.

Data on antenatal steroids were available from 2004 to 2016.

Gestation age was defined according to https://pubmed.ncbi.nlm.nih.gov/23768051/ when the calculation of variation was based on standard deviations (SD), which were calculated from the weight, length, and HC data in all subgroups (SDd).

Ventilator treatment included invasive mechanical ventilation.

Resuscitation was defined as chest compressions and/or intubation performed in the delivery room.

∗ Data on diabetes was available from 1991 to 2004. After 2004, diabetes mellitus was determined using ICD-10 code O24.

Early neonatal mortality risk was markedly greater in infants born MP (OR 10.9, 95% CI 9.0-13.3) and LP (OR 5.9, 95% CI 5.1-6.9) compared with those born full term, although the greatest risk was observed in infants born VP (OR 37.2, 95% CI 32.2-42.9). In the late neonatal period, mortality risks were as follows: infants born LP—OR 1.41, 95% CI 1.08-1.84, infants born MP—OR 0.83, 95% CI 0.54-1.28 and infants born VP—OR 2.56, 95% CI 1.96-3.35. Postneonatal mortality risk did not differ in the MP group (OR 1.10, 95% CI 0.81-1.47), whereas in the infants born LP (OR 1.46, 95% CI 1.22-1.75) it was statistically significantly increased, relative to infants born full term. Infants born VP had the greatest risk of postneonatal mortality (OR 1.45, 95% CI 1.14-1.84).

**Figure 1:**
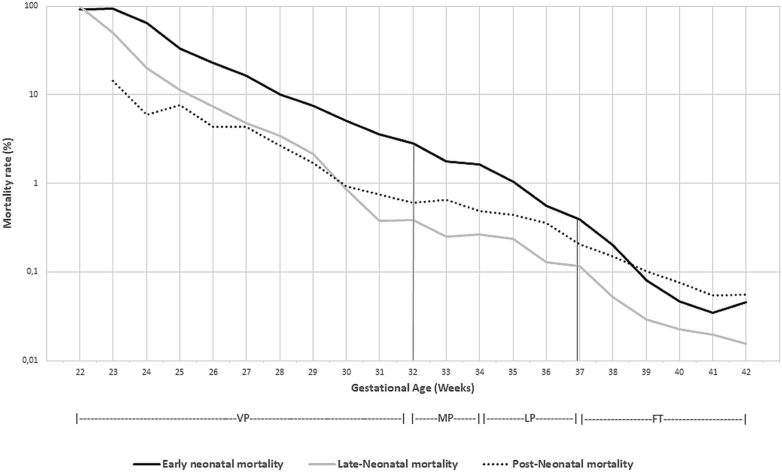
Mortality rates by gestational age at birth for early neonatal (2780 deaths), late neonatal (686 deaths), and postneonatal (1427 deaths) mortality, stillborn and post-term infants excluded (n = 1 465 350). The gestational age at birth is divided into groups: VP, MP, LP, and full term (FT).

Early neonatal, late-neonatal and postneonatal mortality rates by gestational age at birth decreased with increasing gestational weeks (Figure 1). Early neonatal mortality constituted the greatest proportion and late neonatal mortality the lowest proportion of deaths in the infants born MP and LP (Figure 1).

### One-Year Mortality Trends of Live-Born Infants

**Figure 2:**
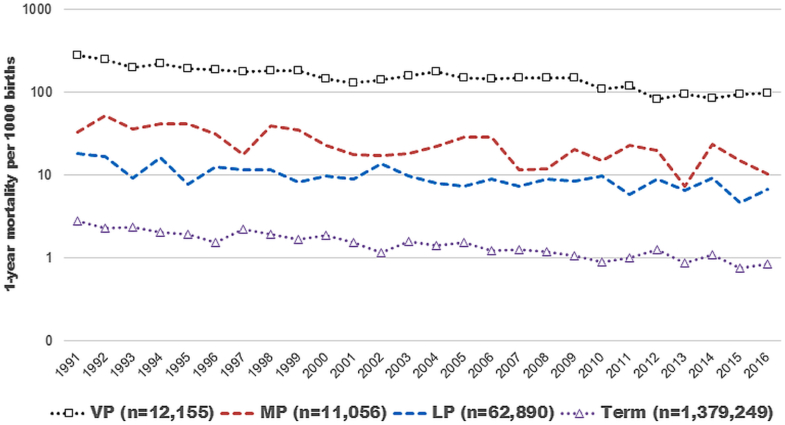
The trend in 1-year mortality per 1000 births from 1991 to 2016 for infants born VP, MP, LP, and FT (n = 1 465 350).

The first-year mortality rate trends in the different gestational age groups during follow-up from 1991 to 2016 are presented in Figure 2. Among the MP group, 1-year mortality decreased from 33 deaths per 1000 births in 1991 to 10 deaths per 1000 births in 2016. Correspondingly, in the LP group, mortality decreased from 18 deaths per 1000 births in 1991 to 7 deaths per 1000 births in 2016. In the analysis on the whole population, mortality during the first year of life decreased statistically significantly by time only in newborns born at full term (Table III). In the LP group, there was a statistically significant decrease in mortality only in the years 2009-2016 compared with the reference period 1991-1995.

**Table III:** Change of 1-year mortality from 1991 to 2016 (total of 4893 deaths among 1 465 350 infants).

Number of infants	VP		MP		LP		FT	
	12 155		11 056		62 890		1 379 249	
Number of deaths	1912 (15.7%)		276 (2.5%)		617 (1.0%)		2088 (0.2%)	
Follow-up periods	IRR	(95% CI)	IRR	(95% CI)	IRR	(95% CI)	IRR	(95% CI)
1991-1995	1.00		1.00		1.00		1.00	
1996-2001	0.81	(0.71-0.92)	0.76	(0.55-1.04)	0.84	(0.67-1.05)	**0.76**	**(0.67-0.85)**
2002-2008	1.23	(1.08-1.41)	0.90	(0.63-1.28)	0.74	(0.59-0.93)	**0.51**	**(0.45-0.57)**
2009-2016	0.91	(0.77-1.09)	0.88	(0.57-1.35)	**0.61**	**(0.47-0.78)**	**0.35**	**(0.31-0.40)**

*FT*, full term.

Models were adjusted for: mother's age, diabetes, smoking after I trimester, primipara, number of fetuses, antenatal steroids, place of birth, mode of delivery, university hospital district, sex of newborn, birth weight for gestational age, Apgar 1 minute, admission to a neonatal unit, resuscitation at birth, ventilator treatment and antibiotic treatment. The results of multivariable-adjusted Poisson regression are shown using incidence rate ratio (IRR) with 95% CIs. Highly statistically significant (*P* < .001) IRRs are shown in bold.

### Predictors of Early Neonatal, Late Neonatal, and Postneonatal Mortality in the Infants Born MP and LP

Among the infants born MP and LP, early neonatal mortality was significantly greater in infants born MP compared with infants born LP, whereas late neonatal and postneonatal mortality did not differ significantly between the groups (Table II). Predictors that were statistically significantly associated with increased mortality risk in all 3 mortality categories were SGA, 1-minute Apgar score of less than 7, and history of ventilator treatment. Birth in one of the university hospital districts seemed to be associated with an increased risk of early neonatal death. Mother's primiparity, twin pregnancy, cesarean delivery, the newborn's admission to a neonatal unit, having received antibiotic treatment, being born in a lower than university level hospital, and being born during the period of 2009-2016 (the period of 1991-1995 being the reference) were associated with a decreased risk of early neonatal death. Mother's smoking during pregnancy had a statistically significant association with an increased risk of postneonatal mortality.

### Causes of Death

The causes of death in the different mortality categories are presented in Table IV. In general, the causes of death in the MP and LP groups were more in accordance with those of the children born at term than those of the children born VP.

**Table IV:** The 3 most common causes of early neonatal, late neonatal, and postneonatal deaths in infants born VP, MP, LP, and at term without post-term and still-born infants (n = 1 465 350)

Mortality	VP (<32^0/7^) n = 12 155	MP (32^0/7^-33^6/7^) n = 11 506	LP (34^0/7^-36^6/7^) n = 62 890	Term (37^0/7^-41^6/7^) n = 1 379 249
Early neonatal (0-6 d)	n = 1470 (0.1%)	n = 188 (1.6%)	n = 348 (0.6%)	n = 774 (0.06%)
I	Short gestation and low birth weight 311 (21%)	Congenital anomalies 144 (77%)	Congenital anomalies 246 (71%)	Congenital anomalies 437 (56%)
II	Respiratory distress of newborn 299 (20%)	Respiratory distress of newborn 11 (5.9%)	Birth asphyxia 13 (3.7%)	Birth asphyxia 75 (0,1%)
III	Intracranial nontraumatic hemorrhage 209 (14%)	Birth asphyxia 9 (4,8%)	Respiratory distress of newborn 12 (3.4%)	Cerebral ischemia, hypoxic ischemic encephalopathy 41 (0.5%)
Late neonatal (7-27 d)	n = 257 (2.1%)	n = 28 (0.2%)	n = 88 (0.1%)	n = 313 (0.02%)
I	Respiratory distress of newborn 60 (23%)	Congenital anomalies 11 (39%)	Congenital anomalies 59 (67%)	Congenital anomalies 190 (61%)
II	Intracranial nontraumatic hemorrhage 54 (21%)	Intracranial nontraumatic hemorrhage <5	Cardiovascular disorders <5	SIDS 22 (7%)
III	NEC 40 (16%)	Bacterial sepsis <5	SIDS <5	Endocrine and metabolic diseases 17 (5.4%)
Postneonatal (28-365 d)	n = 185 (1.5%)	n = 60 (0.5%)	n = 181 (2.9%)	n = 1001 (0.07%)
I	BPD 70 (38%)	Congenital anomalies 27 (45%)	Congenital anomalies 90 (50%)	Congenital anomalies 322 (32%)
II	Congenital anomalies 24 (13%)	SIDS 8 (13%)	SIDS 46 (25%)	SIDS 296 (30%)
III	NEC 17 (9.2%)	Diseases of the nervous system 5 (8.3%)	Endocrine and metabolic diseases 9 (5.0%)	Diseases of the nervous system 85 (8.5%)

*BPD*, bronchopulmonary dysplasia; *NEC*, necrotizing enterocolitis.

Among infants born MP and LP, congenital anomalies were the most common causes of early neonatal mortality. In the MP group, chromosomal anomalies were the leading cause (n = 35, 24%), whereas in the LP group, malformations of the urinary system were the most frequent (n = 76, 31%) cause of early neonatal mortality. For late neonatal and postneonatal mortality, cardiovascular anomalies were most common in the MP group (late neonatal: n = 6, 55%; postneonatal: n = 14, 52%), whereas in the LP group, chromosomal anomalies predominated in late neonatal deaths (n = 22, 37%) and cardiovascular anomalies in postneonatal deaths (n = 35, 39%). Birth asphyxia and respiratory distress of the newborn were most important causes of early neonatal death in the MP and LP groups. Sudden infant death syndrome (SIDS) was the second most common cause of postneonatal death.

## Discussion

This nationwide population-based register study included all children born in Finland during a 25-year period. Our main findings were that the risk of early neonatal mortality was statistically significantly increased in the MP and LP groups compared with the full-term group. The greatest proportion of deaths occurred during the first 6 days of life in all preterm groups, but in the infants born at term, most deaths occurred after the early neonatal period. Early neonatal, late neonatal, and postneonatal mortality rates decreased with increasing gestational age. Among the MP and LP groups, only the risk of early neonatal mortality decreased significantly over time. In the whole population, 1-year mortality decreased significantly over time only in the LP and full-term groups. Some regional differences and differences by hospital level were identified regarding the risk of mortality. Being born SGA, asphyxia at birth, and having received ventilator treatment were associated with an increased risk of mortality in all mortality categories. The causes of death in the MP and LP groups were more in accordance with those in the term group than those of the VP group. Congenital anomalies were the most important causes of death in the MP and LP groups in all mortality categories.

### Mortality Rates

There is a wide variation in perinatal, neonatal, and infant mortality between different countries, with Finland having one of the lowest mortality rates in international comparisons. According to Euro-Peristat,^11^ neonatal mortality rates ranged between 0.5 in 1000 and 4.3 in 1000 births in Europe between 2015 and 2019. During this period, they tended to either decrease or fluctuate at the same level. In addition, infant mortality rates varied between 2015 and 2019 from 3.5 per 1000 to less than 2.0 per 1000 live births. Infants born between 32 and 36 weeks of gestation accounted for approximately 16.5% of neonatal mortality.^11^ In the current study, the greatest proportion of deaths occurred in the live-born infants during the first 6 days of life in all our preterm groups, but in the infants born at term, most deaths occurred after the neonatal period. Similar studies on mortality are scarce. One study from India examined early and late neonatal mortality in infants born preterm and at term but did not report a similar pattern. In that study, 8% of the infants born preterm died during the early neonatal period and 1.0% died during the late neonatal period, whereas among the infants born at term, 2% died early and 0.4% died late, suggesting that most deaths in both the preterm and term groups still occurred during the early neonatal period.^12^

In our study, mortality decreased with advancing weeks of gestation. Accordingly, a secondary analysis of an obstetric cohort of 115 502 women and their nonanomalous live-born neonates in the US between 2008 and 2011 showed a continuum of increasing survival and decreasing morbidity and diagnoses with each advancing week of gestation. A decrease in the frequency of all morbidities was seen at 32 weeks of gestation.^13^

A study conducted in India between 1993 and 2021^14^ studying live-born infants who died before their fifth birthday found that mortality decreased in terms of early neonatal mortality (33.5-20.3 deaths per 1000), late neonatal death (14.1-4.1 deaths per 1000), and postneonatal mortality (31.0-10.8 deaths per 1000).^14^ Mortality during the early neonatal (48.3%) and postneonatal (25.6%) periods contributed to the largest proportion of deaths.^14^

### Mortality Trends

Among the MP and LP groups, a statistically significant decrease in mortality over time was observed only for early neonatal mortality. This might be because the absolute numbers of cases in the late neonatal and postneonatal mortality categories were small. However, significant advances in the care of this population have occurred in perinatal treatment practices, including new recommendations for antenatal corticosteroid treatment.^15^^,^^16^ In addition, improved antenatal screening and new screening recommendations, which were put into practice in Finland in 2010, have probably led to improvements, especially in the detection of cardiac malformations.^17^^,^^18^ Antenatal diagnosis and treatment planning can improve outcomes and reduce mortality.

In our whole population, 1-year mortality rates decreased statistically significantly from the years 1991 to 2016 only in infants born LP and full term. Studies either on similar populations or with similar designs are difficult to find. A study on the differences in singletons born late preterm and at term in the US between 1995 and 2002^6^ found significant decreases in infant mortality rates since 1995 in all age-at-death categories, excluding the late neonatal period.

### Predictors of Mortality

Predictors associated with increased mortality in all mortality categories were SGA, a low Apgar score, and receiving ventilator treatment among the MP and LP groups. These results are in line with earlier studies.

A Canadian population-based cohort study^19^ of approximately 1 700 000 singleton hospital live births at 23-42 weeks of gestation between 2002 and 2015 found that birth with severe SGA was associated with an increased risk of death both in term (adjusted relative risk 38.3, 95% CI 35.4-41.4) and infants born preterm (adjusted relative risk 96.7, 95% CI 85.4-109.5). Another study, from Utah in the US, on approximately 340 000 infants born at gestational ages of 34 weeks or more in 1999-2005 revealed that infants who were SGA born late preterm were 44 times more likely to die in the first month and 22 times more likely to die in the first year compared with infants born appropriate for gestational age. The differences persisted for infants who were SGA, even after the exclusion of lethal congenital anomalies.^20^

Low Apgar scores (0-6) were associated with an increased risk of perinatal, neonatal, and infant mortality in a recent study of approximately 6 800 000 full-term singleton infants born between 2016 and 2017 in the US.^21^ In addition, in a Swedish Medical Birth Register study of 113 300 infants born preterm between 1992 and 2016,^22^ a lower Apgar score was associated with a greater risk of neonatal death in infants born at all gestational ages.

The need for ventilator treatment is obviously a marker of the infant's critical condition and increased risk of death in general. Mechanical ventilation can also cause life-threatening complications, including air leaks and airway injuries.^23^ Preterm delivery increases the risk of respiratory distress syndrome (RDS).^24^ In a retrospective study from the US involving 233 844 deliveries, the odds of RDS decreased with each advancing week of gestation for up to 38 weeks compared with 39-40 weeks. The adjusted OR at 34 weeks was 40.1 (95% CI 32.0-50.3), and at 38 weeks, it was 1.1 (95% CI 0.9-1.4). The same pattern was seen with a decreased need for ventilatory support.^25^ A prospective observational study of 46 neonatal intensive care units from France established management strategies for respiratory failure, including RDS in infants who were MP and LP. It found differences in management and ventilator support practices depending on gestational age despite the same clinical course, which warranted further research on respiratory failure treatment strategies in the moderate preterm and late preterm groups.^26^

Some statistically significant regional differences were found in early neonatal mortality, indicating a need to evaluate treatment practices, quality of care and resources in regions where the risk of early neonatal mortality was increased. Maternal smoking was a risk factor of postneonatal mortality, supporting the fact that smoking during pregnancy should be strongly discouraged.

We found that births in lower than university level hospitals were associated with a lower risk of early neonatal mortality. This might be explained by well-maintained centralization of high-risk pregnancies. The association of modes of delivery and antibiotic use with decreased risk of mortality needs to be explored further, in order to better understand the possible favorable impact.

### Causes of Death

In this study, the causes of death in the MP and LP groups resembled those in the term group rather than in the VP group, for which the most common causes of death were more related to prematurity. Asphyxia and respiratory distress of the newborn were the most important causes of early neonatal mortality in the MP and LP groups, and the proportions of infections were of very low significance. However, a recent study in São Paulo, Brazil, of a population of 545 606 infants born MP and LP without congenital anomalies found that perinatal asphyxia (14%), respiratory disorders (27%), and infections (44%) accounted for most of the causes of death.^27^ The significant proportion of infections in this study seems to be typical of developing countries. A study on 194 countries estimated the causes of death for the early and late neonatal period in 2000-2013 and found that they differed depending on the time of death and the mortality rate of each country.^28^ The most common causes of early neonatal deaths were preterm birth (40.8%) and intrapartum complications (27%). In turn, infections accounted for nearly one-half of the deaths in the late neonatal period. The authors called for more regional studies based on vital registration-based models of this topic and more tailored interventions.^28^

In the current study, SIDS was a significant cause of postneonatal mortality in the MP, LP, and term groups. Prematurity is a known risk factor associated with impaired regulation of the autonomic nervous system.^29^ Counseling parents on their baby's sleeping and bedding habits, etc, to reduce the risk of SIDS needs to be included in the care of infants born MP, LP, and at term.

European Surveillance of Congenital Anomalies, which provides comprehensive information on congenital anomalies in Europe and covers almost all registers of congenital malformations, recorded a total prevalence of major congenital anomalies of 25.5 per 1000 births during 2006-2010.^30^ We found that congenital anomalies were the most common causes of death in all mortality categories in the MP and LP groups and in late and postneonatal mortality categories in the term group. Congenital malformations were the most common cause of all infant deaths (21%) recorded in birth and death certificates in the US in 2017.^31^

A European population-based cohort study from 1995 to 2014 that included 150 198 live-born infants with major congenital anomalies found that preterm birth at 32-36 weeks of gestation was associated with a 3.88 (95% CI 3.40–4.43) risk of death in less than 1 year compared with infants born at term.^32^ Prematurity and low birth weight are also associated with congenital anomalies. According to Dolan et al, infants with birth defects have a significantly increased risk of adverse perinatal outcomes and are 2.7 times more likely to be born before 37 weeks of gestation, 7.0 times more likely to be born before 34 weeks of gestation, and 11.5 times more likely to be born before 32 weeks of gestation. In addition, they were 3.6 times more likely to have birth weight below 2500 g and 11.3 times more likely to weigh less than 1500 g.^33^

Cardiovascular malformation was a major cause of death in our data. According to EUROCAT data from 2006 to 2010, congenital heart defects were the most common subgroup of congenital anomalies, with a prevalence of 8.1 per 1000 births, followed by limb defects (4.1) and chromosomal defects (3.6).^11^ A study of 2189 live births with congenital heart defects conducted in Paris between 2005 and 2008 found that cardiovascular malformation led to a twofold risk of spontaneous preterm birth.^34^

### Strengths and Limitations

This study has several strengths. The long study period allowed us to examine temporal trends in mortality. The extensive data also made it possible to study different mortality categories in different groups of premature births and to seek predictors of mortality among these categories. Additional strengths include the comprehensive data of all births in Finland and the high-quality national health registers.^35^^,^^36^

The limitations of our study are its retrospective design and some missing data. There may also have been some regional, local, or individual variations in the recording practices. In addition, the ICD version changed during the study period, and although we rematched the diagnoses, this change could have led to some bias. For causes of death, we used only death certificate ICD codes, so data on contributing diagnoses were scarce. Also, the cause of death was either determined by a pathologist based on an autopsy or was at the clinician's discretion. Our data collection ended in 2016, which can also be regarded as a limitation. However, the dataset still includes more recent and comprehensive information than much of the previously published literature in this field. Moreover, the dataset is large and required extensive work, which contributed to the time needed for analysis. Although the inclusion of more recent data would undoubtedly provide additional insights into mortality trends after 2016, this is beyond the scope of the present study and will be addressed in future research. Importantly, the absence of post-2016 data does not undermine the validity of the associations and trends observed within the study period.

## Conclusions

Early neonatal, late neonatal, and postneonatal mortality decreased with increased gestational age, and the risk of early neonatal mortality was statistically significantly increased in the MP and LP groups, with the term group being the reference. Among the MP and LP groups, only early neonatal mortality significantly decreased over time, probably due to having benefited from advances in perinatal care. In the whole population first-year mortality was significantly decreased by time only in the LP and term groups. Predictors of increased mortality in all mortality categories in the MP and LP groups were SGA, a low Apgar score, and a history of ventilator treatment. Some differences by region and hospital level in the risk of early neonatal mortality were identified. The causes of death in the MP and LP groups resembled those of the term group more than those of the very preterm group. Congenital anomalies were the most common, albeit not easily preventable, causes of mortality in the MP and LP groups. Improvements focused on the prevention of the second-most common causes, including asphyxia and respiratory distress, can be effective in decreasing early neonatal mortality. Those focused on counseling for the prevention of SIDS can be effective in decreasing late neonatal and postneonatal mortality.

## CRediT authorship contribution statement

**Kati Kallio:** Writing – review & editing, Writing – original draft, Visualization, Validation, Resources, Project administration, Methodology, Investigation, Funding acquisition, Formal analysis, Data curation, Conceptualization. **Riitta Ojala:** Writing – review & editing, Supervision, Methodology, Conceptualization. **Tiina Luukkaala:** Writing – review & editing, Formal analysis, Data curation. **Outi Tammela:** Writing – review & editing, Writing – original draft, Visualization, Validation, Supervision, Resources, Project administration, Methodology, Investigation, Funding acquisition, Conceptualization.

## Declaration of Competing Interest

This study was supported by state research funding via 10.13039/501100010600Tampere University Hospital and the Wellbeing Services County of Pirkanmaa (grant numbers 9AC091 and 9AA074), as well as by The Alma and K. A. Snellman Foundation, Oulu, Finland. The funders had no role in the study. The authors declare no conflicts of interest.
