# Supplementary material for: First-Year Mortality in Infants Born Moderately Preterm and Late Preterm: A National Register Study
Source: J Pediatr Clin Pract. 2026 Mar 24;20:200206. doi: 10.1016/j.jpedcp.2026.200206 (PMC13122826; doi:10.1016/j.jpedcp.2026.200206)
Supplement: Supplementary Table 1 [file mmc1.docx]

Supplementary Table 1. Early neonatal, late neonatal, and post neonatal mortality of live-born children. Multivariable-adjusted logistic regression analyses were used (N=1,465,350). The results are shown by number of deceased infants (n) and odds ratios (ORs) with 95% confidence intervals (CIs). Odds ratios with statistical significance (p < 0.001) are shown in boldface.

|  |  | **Early neonatal mortality**  **(0-6 days)**  n=2,780 (0.2%) of N=1,465,350 | | | **Late neonatal mortality**  **(7-27 days)**  n=686 (0.05%) of N=1,462,570 | | | **Post neonatal mortality**  **(28-365 days)**  n=1,427 (0.1%) of  N = 1,461,884 | | |
| --- | --- | --- | --- | --- | --- | --- | --- | --- | --- | --- |
|  | N | n | OR | (95% CI) | n | OR | (95% CI) | n | OR | (95% CI) |
| **Gestation weeks** |  |  |  |  |  |  |  |  |  |  |
| FT (37^+0^–41^+6^) | 1,379,249 | 774 | 1.00 |  | 313 | 1.00 |  | 1,001 | 1.00 |  |
| VP (<32^+0^) | 12,115 | 1,470 | **37.2** | **(32.2–42.9)** | 257 | **2.56** | **(1.96–3.35)** | 185 | 1.45 | (1.14–1.84) |
| MP (32^+0^–33^+6^) | 11,056 | 188 | **10.9** | **(9.0–13.3)** | 28 | 0.83 | (0.54**–**1.28) | 60 | 1.10 | (0.81**–**1.47) |
| LP (34^+0^–36^+6^) | 62,890 | 348 | **5.9** | **(5.1-6.9)** | 88 | 1.41 | (1.08**–**1.84) | 181 | **1.46** | **(1.22–1.75)** |
| **MOTHER** |  |  |  |  |  |  |  |  |  |  |
| **Age, years**# |  |  |  |  |  |  |  |  |  |  |
| 20–40 | 1,398,720 | 2,559 | 1.00 |  | 638 | 1.00 |  | 1,291 | 1.00 |  |
| <20 | 36,088 | 94 | 1.36 | (1.07–1.73) | 25 | 1.56 | (1.02**–**2.38) | 84 | **2.38** | **(1.88–3.02)** |
| >40 | 30,540 | 127 | 1.41 | (1.14–1.74) | 23 | 1.03 | (0.67**–**1.58) | 52 | 1.33 | (1.00**–**1.77) |
| **Diabetes*** |  |  |  |  |  |  |  |  |  |  |
| No | 1,389,824 | 2,660 | 1.00 |  | 657 | 1.00 |  | 1,357 | 1.00 |  |
| Yes | 75,526 | 100 | 0.80 | (0.64–0.99) | 29 | 0.79 | (0.53**–**1.16) | 70 | 0.92 | (0.72**–**1.18) |
| **Smoking after I trimester** |  |  |  |  |  |  |  |  |  |  |
| Nonsmoker | 1,210,722 | 2,095 | 1.00 |  | 544 | 1.00 |  | 1,008 | 1.00 |  |
| Quit | 50,155 | 67 | 1.04 | (0.80–1.36) | 16 | 0.89 | (0.53**–**1.47) | 47 | 1.32 | (0.98**–**1.78) |
| Smoked | 169,748 | 384 | 0.91 | (0.80–1.03) | 95 | 0.94 | (0.75**–**1.18) | 334 | **1.83** | **(1.61–2.08)** |
| Missing | 34,725 | 234 | **1.85** | **(1.56–2.19)** | 31 | 1.23 | (0.84**–**1.79) | 38 | 1.06 | (0.76**–**1.47) |
| **Primipara** |  |  |  |  |  |  |  |  |  |  |
| No | 872,141 | 1,601 | 1.00 |  | 395 | 1.00 |  | 901 | 1.00 |  |
| Yes | 592,350 | 1,148 | **0.65** | **(0.60–0.71)** | 291 | **0.69** | **(0.58–0.81)** | 542 | **0.60** | **(0.53–0.67)** |
| Unknown | 859 | 31 | **3.67** | **(2.04–6.57)** | 0 | **-** |  | 2 | 1.32 | (0.32**–**5.43) |
| **Number of fetuses** |  |  |  |  |  |  |  |  |  |  |
| 1 | 1,420,499 | 2243 | 1.00 |  | 575 | 1.00 |  | 1,334 | 1.00 |  |
| 2 | 43,738 | 498 | 1.06 | (0.94–1.19) | 107 | 1.17 | (0.93**–**1.46) | 89 | 0.70 | (0.55–0.87) |
| ≥3 | 1,113 | 39 | 1.46 | (0.99–2.16) | 4 | 0.63 | (0.23**–**1.71) | 4 | 0.54 | (0.20**–**1.45) |
| **Antenatal steroids** |  |  |  |  |  |  |  |  |  |  |
| No | 1,446,238 | 2,469 | 1.00 |  | 587 | 1.00 |  | 1,322 | 1.00 |  |
| Yes | 19,112 | 311 | **0.66** | **(0.56–0.78)** | 99 | 1.43 | (1.08**–**1.91) | 105 | 1.48 | (1.16**–**1.89) |
| **Place of birth**# |  |  |  |  |  |  |  |  |  |  |
| University hospital | 487,487 | 1,753 | 1.00 |  | 416 | 1.00 |  | 672 | 1.00 |  |
| Central hospital | 679,967 | 810 | 0.89 | (0.80–0.98) | 199 | **0.72** | **(0.59–0.87)** | 534 | **0.79** | **(0.70–0.90)** |
| Other | 297,893 | 215 | 0.95 | (0.80–1.11) | 71 | 1.15 | (0.86**–**1.54) | 221 | 0.90 | (0.76**–**1.07) |
| **Mode of delivery** |  |  |  |  |  |  |  |  |  |  |
| Vaginal | 1,223,218 | 1,556 | 1.00 |  | 360 | 1.00 |  | 971 | 1.00 |  |
| Elective cesarean delivery | 110,894 | 255 | 0.81 | (0.69–0.95) | 88 | 1.19 | (0.93**–**1.53) | 144 | 1.10 | (0.91**–**1.32) |
| Nonelective caesarean delivery | 130,407 | 942 | **0.71** | **(0.64-0.78)** | 236 | 0.96 | (0.79-1.15) | 312 | 1.01 | (0.87-1.17) |
| Missing | 831 | 27 | 2.53 | (1.35–4.72) | 2 | 2.57 | (0.59**–**11.2) | 0 | - |  |
| **University Hospital District**# |  |  |  |  |  |  |  |  |  |  |
| A | 506,052 | 907 | 1.00 |  | 237 | 1.00 |  | 516 | 1.00 |  |
| B | 188,149 | 335 | 1.10 | (0.95–1.27) | 64 | 0.65 | (0.49**–**0.87) | 205 | 0.91 | (0.77**–**1.08) |
| C | 324,760 | 692 | **1.61** | **(1.43–1.81)** | 193 | **1.42** | **(1.16–1.74)** | 270 | 0.82 | (0.70**–**0.95) |
| D | 215,945 | 404 | **1.32** | **(1.15–1.52)** | 95 | 0.91 | (0.70**–**1.17) | 197 | 0.81 | (0.68**–**0.96) |
| E | 230,229 | 438 | 1.18 | (1.03–1.35) | 97 | 0.77 | (0.60**–**0.98) | 238 | 0.85 | (0.72**–**0.99) |
| **NEWBORN** |  |  |  |  |  |  |  |  |  |  |
| **Sex** |  |  |  |  |  |  |  |  |  |  |
| Boy | 748,482 | 1,572 | 1.00 |  | 375 | 1.00 |  | 816 | 1.00 |  |
| Girl | 716,868 | 1,208 | 0.89 | (0.81–0.97) | 311 | 0.80 | (0.84**–**1.14) | 611 | 0.85 | (0.76**–**0.94) |
| **Birth weight for gestational age** |  |  |  |  |  |  |  |  |  |  |
| AGA | 1,387,953 | 2,298 | 1.00 |  | 550 | 1.00 |  | 1,212 | 1.00 |  |
| SGA | 37,590 | 370 | **4.56** | **(3.97–5.23)** | 116 | **4.27** | **(3.43–5.31)** | 189 | **3.61** | **(3.07–4.25)** |
| LGA | 39,807 | 112 | **1.58** | **(1.27–1.96)** | 20 | 1.10 | (0.70**–**1.74) | 26 | 0.59 | (0.40**–**0.88) |
| **Apgar 1 minute** |  |  |  |  |  |  |  |  |  |  |
| 7–10 | 1,391,513 | 472 | 1.00 |  | 297 | 1.00 |  | 1,014 | 1.00 |  |
| 0–6 | 71,063 | 2,213 | **29.7** | **(26.4–33.5)** | 380 | **3.69** | **(3.02–4.50)** | 399 | **2.37** | **(2.05–2.76)** |
| Missing | 2,774 | 95 | **32.1** | **(24.6-42.0)** | 9 | **3.89** | **(1.96–7.73)** | 14 | **2.65** | **(1.54–4.57)** |
| **Admission to a neonatal unit** |  |  |  |  |  |  |  |  |  |  |
| No | 1,317,643 | 1,088 | 1.00 |  | 167 | 1.00 |  | 724 | 1.00 |  |
| Yes | 147,707 | 1,692 | 0.81 | (0.72–0.93) | 519 | **6.50** | **(5.05–8.37)** | 703 | **3.84** | **(3.29–4.49)** |
| **Resuscitation at birth** |  |  |  |  |  |  |  |  |  |  |
| No | 1,452,829 | 1,584 | 1.00 |  | 425 | 1.00 |  | 1,240 | 1.00 |  |
| Yes | 12,521 | 1,196 | **3.92** | **(3.50–4.40)** | 261 | **2.62** | **(2.13–3.229** | 187 | 1.39 | (1.14**–**1.70) |
| **Ventilator treatment** |  |  |  |  |  |  |  |  |  |  |
| No | 1,447,608 | 1,615 | 1.00 |  | 330 | 1.00 |  | 1,099 | 1.00 |  |
| Yes | 17,472 | 1,165 | **2.32** | **(2.00-2.68)** | 356 | **5.02** | **(3.84–6.55)** | 338 | **4.01** | **(3.25–4.95)** |
| **Antibiotic treatment** |  |  |  |  |  |  |  |  |  |  |
| No | 1,399,390 | 1,908 | 1.00 |  | 344 | 1.00 |  | 1,019 | 1.00 |  |
| Yes | 65,960 | 872 | **0.21** | **(0.18–0.24)** | 342 | 0.83 | (0.66**–**1.04) | 408 | 1.03 | (0.86**–**1.23) |
| **Study period** |  |  |  |  |  |  |  |  |  |  |
| 1991–1995 | 307,193 | 881 | 1.00 |  | 192 | 1.00 |  | 416 | 1.00 |  |
| 1996–2001 | 329,381 | 702 | 0.86 | (0.70–0.83) | 198 | 0.95 | (0.77**–**1.16) | 356 | **0.77** | **(0.66–0.88)** |
| 2002–2008 | 383,027 | 669 | **0.76** | **(0.67–0.86)** | 157 | **0.52** | **(0.42–0.66)** | 348 | **0.58** | **(0.50–0.67)** |
| 2009–2016 | 445,749 | 528 | **0.55** | **(0.48–0.64)** | 139 | **0.39** | **(0.30–0.50)** | 307 | **0.43** | **(0.37–0.51)** |
|  |  |  |  |  |  |  |  |  |  |  |

VP=very preterm, MP=moderate preterm, LT=late preterm, FT=Full term; #=number of the unknowns <1% in all term groups, not presented separately. Highly statistically significant (p<0.001) ORs were shown in **bold**face.
